# Investigating the Anticancer Effects of Sulforaphane in an In Vitro Coculture Model of Prostate Cancer Cells with Engineered Heart Tissue

**DOI:** 10.1021/acsptsci.5c00622

**Published:** 2025-11-14

**Authors:** Jane In den Birken, Laura Rathjens, Hannah Münch, Tina Rohlfing, Konstantina Stathopoulou, Alexandra Rhoden, Gunhild von Amsberg, Thomas Eschenhagen, Sergey Dyshlovoy, Friederike Cuello

**Affiliations:** † Institute of Experimental Pharmacology and Toxicology, 37734 University Medical Center Hamburg-Eppendorf, Martinistrasse 52, 20246 Hamburg, Germany; ‡ DZHK (German Center for Cardiovascular Research), Partner Site Hamburg/Kiel/Lübeck, University Medical Center Hamburg-Eppendorf, Martinistrasse 52, 20246 Hamburg, Germany; § Department of Oncology, Hematology and Bone Marrow Transplantation with Section Pneumology, Hubertus Wald Tumorzentrum—University Cancer Center Hamburg (UCCH), University Medical Center Hamburg-Eppendorf, 20246 Hamburg, Germany; ∥ Martini-Klinik, Prostate Cancer Center, University Medical Center Hamburg-Eppendorf, Martinistrasse 52, 20246 Hamburg, Germany; ⊥ Laboratory of Biologically Active Compounds, Institute of Science-Intensive Technologies and Advanced Materials, Far Eastern Federal University, 690922 Vladivostok, Russian Federation

**Keywords:** prostate cancer, hiPSC-cardiomyocytes, cardiotoxicity, sulforaphane, in vitro coculture, engineered heart tissue

## Abstract

Sulforaphane (SFN) is a phytoderived compound abundant in cruciferous plants that possesses a broad spectrum of anticancer properties. We showed that SFN-induced caspase-mediated apoptosis in grade IV bone metastasis-derived androgen-insensitive PC-3 (IC_50_ = 4.2 μM), and lymph node metastasis-derived androgen-sensitive LNCaP (IC_50_ = 2.8 μM) prostate adenocarcinoma cells. SFN-mediated cardiotoxic side effects were tested in a preclinical in vitro model that enables the study simultaneously of the impact of drugs on cancer cell death and contractile properties of engineered heart tissues generated from human-induced pluripotent stem cell-derived cardiomyocytes (hiPSC-CM EHT). Thereby, SFN exposure induced PC-3 cell death without affecting the contractile force of hiPSC-CM EHT. Interestingly, the irregular beating pattern of hiPSC-CM EHT observed in the presence of PC-3 coculture was normalized compared to vehicle treatment. Overall, this in vitro coculture model of hiPSC-CM EHT and cancer cells could facilitate the study of cardiotoxic cancer drug side-effects.

## Graphical abstract

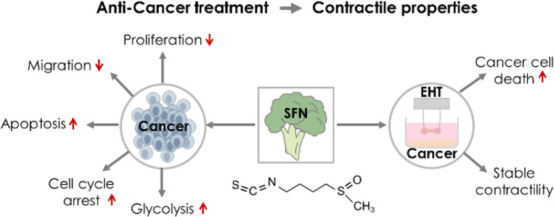

Sulforaphane (SFN) is an isothiocyanate (ITC) abundant in cruciferous plants, such as broccoli or cauliflower. The presence of its N=C=S functional group, a characteristic element of the ITC family, is the key factor that conveys biological activity of SFN facilitating formation of a covalent bond between SFN and cellular proteins.^1^ Mechanistically, a positively charged carbon atom acts as an electrophile to form an adduct with nucleophilic groups, such as thiolates in cysteine residues of target proteins. This post-translational modification can modulate the activity of cellular proteins by blocking oxidation sites and thus preventing the formation of disulfide bridges via cysteine residues in proteins. In consequence, SFN-modification of proteins enables activation or inhibition of key transcription factors such as Kelch-like ECH-associated protein (KEAP1)/nuclear factor erythroid 2 (Nrf2) or nuclear factor kappa B.^2–4^ Due to its broad range of reported beneficial actions in the treatment of various cancer types, neurological disorders, obesity, and inflammatory diseases,^5,6^ SFN had also been successfully tested as a therapy for prostate cancer (clinicalTrials.gov as NCT01959143) that unveiled its therapeutic inhibition of cancer progression.^7^

Previously, we reported that administration of SFN to engineered heart tissue (EHT) impaired stretch-induced enhancement of force production in cardiomyocytes, also known as the Frank-Starling response, as a potential cardiotoxic side effect.^8^ In the present study, we further explored the SFN effects on cardiomyocytes cocultured with prostate cancer cells. This coculture model allows simultaneous examination of anticancer effects of SFN as well as monitoring potential effects on cardiomyocyte contractile properties.

## Materials and Methods

All chemicals, dimethyl sulfoxide (DMSO #8418), SFN (#S4441), fibrinogen (#F8630), thrombin (#605157), insulin (#I9278), aprotinin (#A1153), tranexamic acid (#857653), hydrocortisone (#H0888), triiodothyronine T3 (#IRMM469), and DMEM low glucose (#D5546) were purchased from Sigma-Aldrich (Taufkirchen, Germany) if not stated otherwise. Y-27632 (#orb60104) was from Biorbyt (Cambridge, UK). RPMI 1640 Glutamax (#61870044), fetal calf serum (FCS, #10270106), penicillin/streptomycin (#15140122), trypsin–EDTA (#15400054) were from Thermo Fisher Scientific (Waltham, MA, USA). NucView 488 Caspase-3 Substrate (#10402) was from Biotinum (Fremont, CA, USA).

### Human Prostate Cell Lines

PNT2 human prostate epithelium cells, as well as PC-3 and LNCaP human prostate cancer cells, were purchased from ATCC (Manassas, VA, USA). The cells were cultured in RPMI Glutamax, 10% (v/v) FCS, and 1% (v/v) penicillin/streptomycin at 37 °C and 7% CO_2_.

### Proliferation Assay

PNT2, PC-3, and LNCaP cells were seeded in 6-well plates with 10^4^ cells in 2 mL per well. After 1, 3, or 5 days of incubation in a drug-free medium, the cells were treated with vehicle at noncytotoxic concentrations (dimethyl sulfoxide, DMSO; 0.1%) or SFN (0.1 μM, 0.3 μM, 1 μM, 3 μM, 10 μM, and 30 μM). After 7 days, the medium was collected, and the cells were detached using trypsin. Cells and medium were centrifuged at 300*g* for 5 min. The supernatant was removed, and cells were resuspended in 1 mL of medium. Viable cells were counted automatically using a semiautomated cell counter (Beckman Coulter Vi-CELL; Beckman Coulter, Krefeld, Germany) and by trypan blue cell staining of the dead cells. Nonstained cells were considered viable. Upon the results obtained from these experiments, 3 μM SFN was used for long-term and 30 μM for subsequent short-term experiments.

### Analysis of Cell Cycle Progression and Apoptosis

PNT2, PC-3, and LNCaP cells were seeded in 6-well plates with 2.5 × 10^4^ cells per well and cultured overnight. Cells were treated with vehicle (DMSO; 0.1%), SFN (1 μM, 3 μM, 10 μM, and 30 μM), or cisplatin (10 μM) for 72 h and detached using trypsin. Cells were fixed in 70% ethanol/H_2_O (v/v) and stored at −20 °C for 48 h. The fixation solution was discarded, and cells were stained using propidium iodide (PI)/RNase buffer. Assessment of cell cycle progression and number of apoptotic cells was performed using fluorescence-activated cell sorting (FACS Calibur; BD Bioscience, San Jose, CA, USA) followed by quantification (BD Bioscience Cell Quest Pro v.5.2.1. software). Apoptotic cells (cells possessing fragmented DNA) were detected as a sub-G1 population.

### Caspase-3 Assay

Investigation of SFN-induced cancer cell apoptosis in PC-3 cells was performed using the NucView 488 Caspase-3 substrate assay (Biotinum, Fremont, CA, USA). Precisely, 10^4^ PC-3 cells were seeded per well of a 96-well plate and incubated for 3 h, and then either vehicle (DMSO; 0.1%) or SFN was added to the medium. To assess short- or long-term effects of SFN, the cells were incubated with 3 μM SFN for 48 h or 30 μM SFN for 1 h, respectively. These SFN concentrations were chosen from the concentration–response experiments performed in Figure 1. The medium was removed 30 min before the end of the experiment, and 50 μL of fresh medium containing NucView substrate (5 μM) was added to the cells. Brightfield and fluorescence images were acquired with an EVOS Cell Imaging Systems microscope (Thermo Fisher Scientific, Waltham, MA, USA). Apoptotic cells were identified by NucView 488 Caspase-3 substrate fluorescence visualized with a GFP filter.

**Figure 1:**
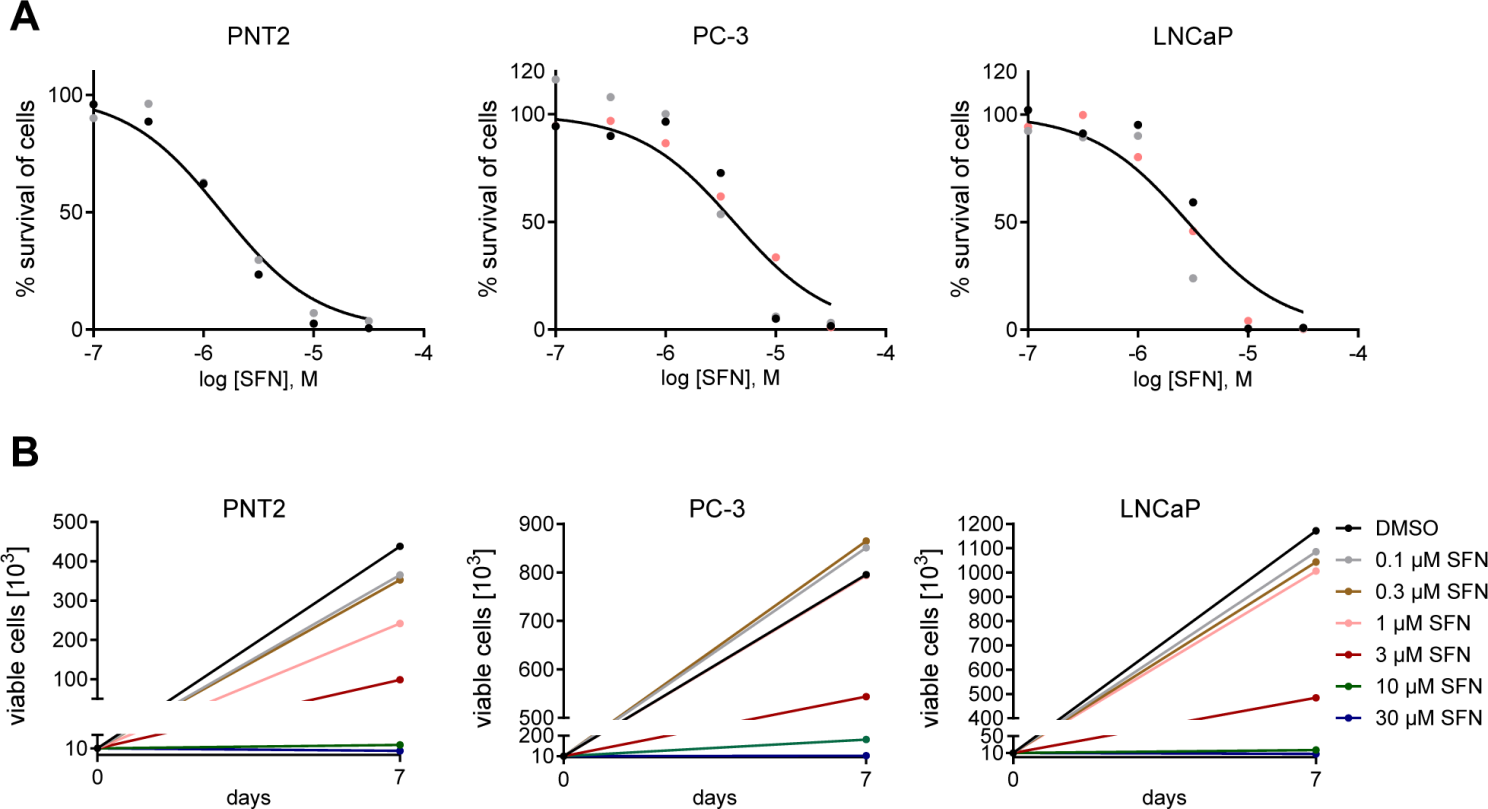
Impact of SFN on prostate epithelial and cancer cell proliferation: noncancer PNT2 prostate epithelial cells, and prostate cancer PC-3 or LNCaP cells were exposed for 7 days to increasing concentrations of sulforaphane (SFN; 0.1–30 μM) or vehicle (DMSO; 0.1%). Cell count was analyzed using a trypan blue-based assay, and IC_50_ values were calculated and visualized graphically as (A) % survival at logarithmic SFN concentration or (B) number of viable cells. The assay was performed in repeated measures with matched values at 0 and 7 days of SFN exposure. Each differentially colored dot represents a separate cell batch. Please note the different scale of the *y*-axis in B between cell lines. PNT2:2 batches, PC-3 and LNCaP: 3 batches; at least 3 technical replicates per batch.

### Colony Formation Assay

For colony formation assays, 1.5 × 10^4^ PC-3 cells per well were seeded to a 6-well plate and incubated overnight. Then, the cells were exposed to vehicle (DMSO; 0.1%) or SFN (3 μM) added to the culture medium for 7 days with daily medium exchange. At day 7, cells from each treatment group were detached by trypsin–EDTA, counted, and reseeded into 6-well plates at a density of 100 cells/well supplemented with 3 mL of medium per well. Cells were further cultured for 12 days (37 °C, 5% CO_2_) without medium change and afterward fixed for 25 min at room temperature (RT) with 1 mL methanol per well. Subsequently, methanol was removed, and wells were air-dried. Giemsa aqueous solution (1 mL/well of 10% (w/v)) was added per well and incubated for 25 min at RT to stain colonies. Excess stain was removed by washing the wells with double-distilled H_2_O. The plates were further air-dried, scanned, and the colonies were counted using a CanonScan LiDE200 scanner (Tokyo, Japan).

### Scratch Assay

For the scratch assay, PC-3 cells were plated in plastic cell culture dishes containing a removable polymer cross with 1.5 × 10^4^ cells per compartment. Cells were treated with either SFN (3 μM) or vehicle for 3 days or SFN (30 μM) or vehicle (DMSO; 0.1%) for 1 h. Then, the polymer cross was removed, and pictures were taken at different time points (0, 16, 24, and 40 h). The relative cell-free area was calculated using ImageJ.^9^

#### 2-Desoxy-2-[(7-nitro-2,1,3-benzoxadiazol-4-yl)­amino]-d-glucose (2-NBDG)-Uptake Assay

PC-3 cells (10^5^ cells per well) were plated into a 6-well plate and incubated overnight. Then, the medium was exchanged to glucose-free medium containing 0.1% FBS. Cells were treated with either SFN (1 μM, 3 μM, and 10 μM), the corresponding vehicle control (DMSO; 0.1%) or phloridzin (100 μM), phloretin (25 μM), or cytochalasin B (5 μM) for 24 h. Afterward, 50 μM 2-NBDG solution (SelleckChem) or vehicle (ethanol; 0.1%) was added, and cells were incubated for 30 min and further stained with PI. The uptake of 2-NBDG by intact (PI-negative) cells was determined by FACS (Calibur, BD Bioscience, San Jose, CA, USA) followed by quantification using BD Bioscience Cell Quest Pro v.5.2.1. software.

### Human-Induced Pluripotent Stem Cell-Derived Cardiomyocytes

HiPSC-CMs were differentiated from an in-house hiPSC control line (UKEi001-A), registered at the Human Pluripotent Stem Cell Registry (hPSCreg.eu) approved by the Ethical Committee of the University Medical Center Hamburg-Eppendorf (Az. PV4798, 28.10.2014) with written informed consent. Differentiation was performed as previously described.^10,11^

### Generation of Engineered Heart Tissue and Contraction Analysis

EHT was generated from hiPSC-CMs as previously described.^10^ In brief, a reconstitution mix containing 10^6^/mL hiPSC-CMs, 5 mg/mL fibrinogen, 3 U/mL thrombin, and 2× DMEM (DMEM complemented with 20% horse serum, 2% penicillin/streptomycin) matching the volume of ingredients without nutrients was prepared. For hiPSC-CM-derived EHT, the Rho-kinase inhibitor Y-27632 (1 μM) was added to avoid cell death during casting. For each EHT, 97 μL of the reconstitution mix was separately added to 3 μL thrombin and pipetted into agarose (2% (w/v) in PBS) casting molds with PDMS racks (C0001; DiNABIOS Deutschland GmbH, Germany) in a 24-well plate. EHT were then incubated (37 °C, 7% CO_2_, 40% O_2_) until fibrin polymerization, before they were transferred to a new, medium-filled culture plate and kept in culture. Culture medium (DMEM low glucose with 10% (v/v) horse serum, Glutamax/Glutamine 200 mM, 1% (v/v) penicillin/streptomycin, 10 μg/mL insulin, 33 μg/mL aprotinin, 200 μM tranexamic acid) was exchanged every other day.

Auxotonic contractions were measured with a video-optical system (EHT Technologies GmbH, Germany) as previously described 2 h after medium exchange.^12^ A custom-made software program (Consulting Team Machine Vision, CTMV, Pforzheim, Germany) automatically detects individual EHT and calculates contraction parameters. To compare the contractile function of EHT between intervention groups under frequency-controlled conditions, EHT were paced electrically using a Grass Astro-Med stimulator model S88X Dual Output Square Pulse Stimulator with an output voltage of 2 V in biphasic pulses of 4 ms. This way, EHT was synchronized at increasing frequencies (0–4 Hz in 0.5 Hz steps) to allow comparison of developed force under different treatment conditions or to induce stress conditions at higher frequencies.

### Pharmacological Intervention of EHT

HiPSC-CM EHT was exposed to vehicle (DMSO; 0.1%) or SFN (3 μM). Two days prior to treatment start, hiPSC-CM EHT was transferred to serum-free medium by replacing the serum with hydrocortisone (50 ng/mL) and triiodothyronine T3 (0.5 ng/mL). During the pharmacological intervention, the medium was exchanged daily. During the treatment, contractile force measurements were performed daily.

### Statistical Analyses

Data were expressed as the mean ± SEM. EHT and batch numbers as well as the individual statistical test were indicated in each figure legend. Statistical analyses were performed with GraphPad Prism 10 software (GraphPad Software Inc. San Diego, CA, USA). A value of *P* < 0.05 was considered significant.

## Results and Discussion

### Antiproliferative Effect of SFN on Human Prostate Cancer Cells

To investigate the short- and long-term anticancer effects of SFN on prostate epithelial and cancer cells, 2D-cultured PNT2 prostate epithelium, grade IV bone metastasis-derived androgen-insensitive PC-3 cells, and lymph node metastasis-derived androgen-sensitive LNCaP prostate adenocarcinoma cells were treated with vehicle (DMSO) or increasing SFN concentrations (1 μM, 3 μM, 10 μM, and 30 μM) for 7 days. Viable and dead cells were distinguished by trypan blue staining and counted to calculate IC_50_ values (Figure 1A,B). SFN could significantly reduce the cell number in a concentration-dependent manner in all three cell lines tested at low micromolar concentrations (PNT2: IC_50_ = 1.7 μM, *n* = 6; PC-3: IC_50_ = 4.2 μM, *n* = 9; LNCaP: IC_50_ = 2.8 μM: *n* = 9).

### Pro-Apoptotic Effects of SFN

Subsequently, the impact of SFN on apoptosis and cell cycle progression of prostate cancer cells was analyzed (Figure 2A,B). PNT2, PC-3, and LNCaP cells were treated for 72 h with vehicle (DMSO), with increasing concentrations of SFN or cisplatin. In this experiment, cisplatin, an established anticancer drug that is frequently used in chemotherapy to treat various cancers in patients, including aggressive variants of prostate cancer, was used as a positive control.^13^ The apoptotic cells identified by DNA fragmentation and the cell cycle phase distribution were analyzed by flow cytometry (Figure 2A,B). For all three cell lines, the percentage of apoptotic cells significantly increased after exposure to 30 μM SFN compared to vehicle-treated cells (PNT2:48.47% ± 7.35%; PC-3:18.40% ± 5.31%; LNCaP: 31.89% ± 3.65%, *n* = 3; Figure 2A). LNCaP was most sensitive to SFN exposure, as apoptosis induction was detected already at 10 μM SFN. The apoptosis was accompanied by a pronounced SFN-induced arrest in cell cycle progression in PNT2, PC-3, and LNCaP cells. Of note, following exposure to 10 μM SFN, the cell cycle of PNT2, PC-3, and LNCaP cells was arrested in the G2/M phase (Figure 2B), while in PC-3 prostate adenocarcinoma cells, a significant proportion of cells remained in the S-phase (Figure 2B; middle), explaining the lower susceptibility of PC-3 cells to SFN and cisplatin (Figure 1A,B).

**Figure 2:**
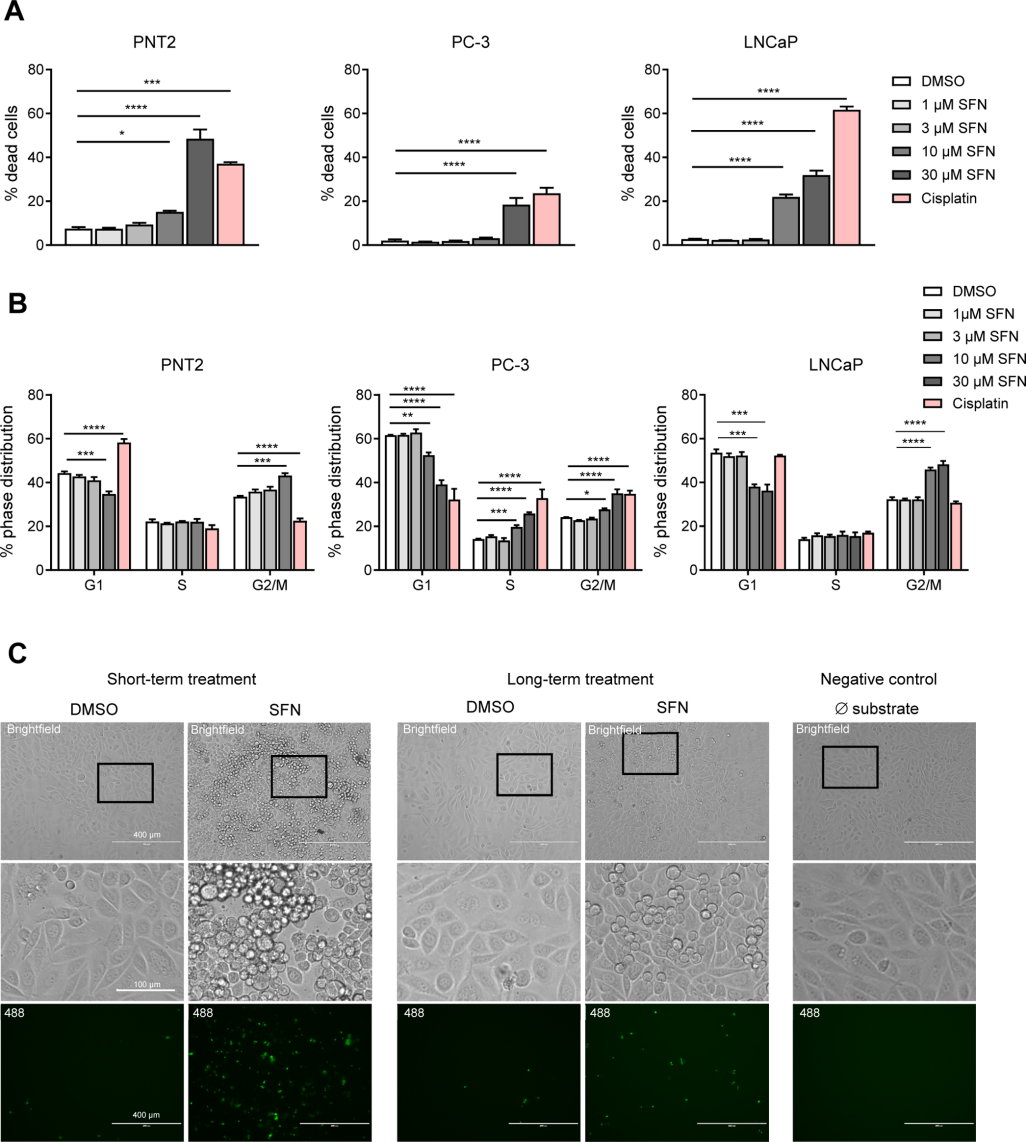
Impact of SFN on cancer cell death and cell cycle progression: (A) PNT2 (*n* = 3), PC-3 (*n* = 3), or LNCaP cells (*n* = 3) was treated for 72 h with SFN (0.1 μM, 0.3 μM, 1 μM, 3 μM, 10 μM, and 30 μM), vehicle (DMSO; 0.1%), or cisplatin (10 μM) as positive control and analyzed for DNA fragmentation (appeared as sub-G1 population) and (B) cell cycle distribution using flow cytometry technique. Data are shown as mean ± SEM. Statistical analysis was performed using one-way ANOVA with Dunnett’s *post hoc* test versus the respective control group. (C) PC-3 cells were treated with vehicle (DMSO; 0.1%) or SFN either long-term with 3 μM SFN for 48 h or short-term with 30 μM for 1 h, and the caspase-3 activity was analyzed with a NucView 488 Caspase-3 assay. The boxes indicate areas that were magnified and shown in the middle images. Scale bar at top and bottom images: 400 nm. Scale bar in the middle images: 100 μm *P < 0.05, **P < 0.01, ***P < 0.001, and ****P < 0.001.

Interestingly, in some cell lines, the effect of cisplatin on cell cycle progression was distinct from that of SFN (Figure 2B). This phenomenon may be related to the mechanism of cisplatin action, which forms covalent DNA cross-links that trigger apoptosis and cell type-specific cell cycle arrest,^13^ whereas SFN primarily acts by covalently binding to specific cellular proteins, thereby modulating transcription factor activity.^1^

We further investigated the potential contribution of caspases in SFN-induced apoptosis. PC-3 cells were exposed to SFN either short-term (30 μM; 1 h) or long-term (3 μM; 48 h) and further analyzed by a NucView 488 Caspase-3 assay (Figure 2C). Compared to vehicle (DMSO) control cells, exposure to SFN resulted in increased nuclei fluorescence, indicating enhanced caspase activity in the nucleus of SFN-treated PC-3 cells. This effect was most pronounced in cells that were treated with SFN for short-term (Figure 2C; upper panels). Furthermore, cells exposed to SFN for short- or long-term exposure showed a distinct morphology compared to those treated with vehicle. Under vehicle-treatment conditions, PC-3 cells exhibited a fusiform shape while exposure to SFN-induced cell rounding and detachment from the culture substrate (Figure 2C; brightfield images).

### Effects of SFN on Colony Formation of Cancer Cells

We investigated the effect of SFN modulating the ability of individual cancer cells to form colonies. This model is assumed to represent a surrogate for metastasis spread and growth in vivo. At the same time, it should be noted that this model possesses important limitations comprising the absence of several essential determinants of metastatic dissemination and colonization, such as the lack of 3D structure, extracellular matrix, stromal and immune context, cell motility, and vascular interactions.^14^ In our experiments, PNT2, PC-3, and LNCaP cells were treated either short- (30 μM, 1 h; Figure 3A; upper panels) or long-term (3 μM, 7 days; Figure 3A; bottom panels) with vehicle (DMSO; white bars) or SFN (gray bars). Short-term exposure to SFN significantly reduced the number of colonies formed by PNT2 and LNCaP cells but did not impact the ability of PC-3 cells to form colonies, corroborating again the lower susceptibility observed before (Figure 1A–C). Long-term exposure to a lower concentration of SFN (3 μM) markedly suppressed the colony formation capacity of PNT2 and PC-3 cells, but not of LNCaP cells compared to the vehicle control (Figure 3A,B; bottom panels).

**Figure 3:**
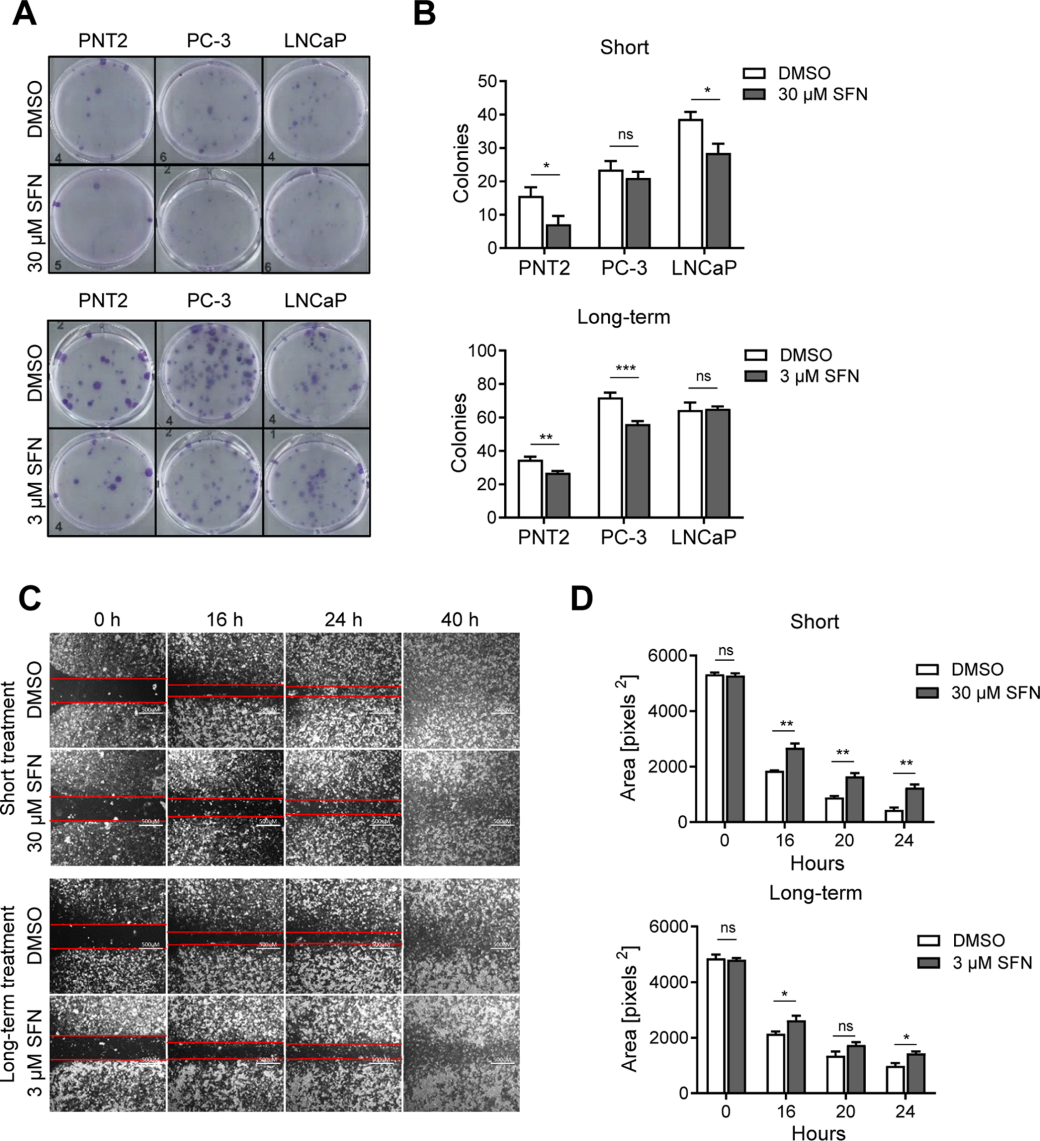
Impact of SFN on prostate epithelial and cancer cell motility. (A,B) Colony formation assay. PNT2, PC-3, or LNCaP cells was treated either short-term (*n* = 9; top panel) with 30 μM SFN (gray bars) for 1 h or long-term (*n* = 6; bottom panel) with 3 μM SFN (gray bars) for 7 days or vehicle (DMSO; 0.1%; white bars). 100 alive cells were then plated, incubated for 12 days, fixed, stained with Giemsa, and formed colonies were counted by eye. (A) Representative images of cell culture dishes (left panels). (B) Quantification of formed-colonies are represented by the bar charts. (C,D) Scratch assays. PC-3 cells were treated either short-term (*n* = 4; top panel) with 30 μM SFN (gray bars) for 1 h or long-term (*n* = 4; bottom panel) with 3 μM SFN (gray bars) for 3 days or vehicle (DMSO; 0.1%; white bars) in cell culture dishes with a polymer cross inlay. After the polymer cross was removed, pictures were taken at the indicated time points (16 h, 24 h, and 40 h), and the relative cell-free area was calculated. (C) Representative images of cell culture dishes (left panels). (D) Cell-free area was quantified and shown as bar charts. Data are shown as mean ± SEM. Statistical analysis was performed using unpaired two-tailed *t*-test per cell type or time point. *P < 0.05, **P < 0.01, and ***P < 0.001.

To explore the effect of SFN on cancer cell motility, wound scratch assays were performed on PC-3 cells. Cells were cultured in subdivided culture dishes that were separated into compartments by a removable polymer cross. After exposure to SFN, either short-term (30 μM, 1 h; Figure 3C; top panels) or long-term (3 μM, 3 days; Figure 3C; bottom panels), the polymer cross was removed, and the time-dependent closure of the cell-free area was evaluated as a surrogate of cell motility. Exposure to SFN revealed a significant reduction in PC-3 cell motility compared to vehicle treatment (relative increase in cell free area after 24 h: short-term: +180.86% Figure 3D; top panels, *n* = 4; long-term: +45.17%; Figure 3D; bottom panels, *n* = 4).

### Stimulation of Glucose Uptake and Glycolysis by SFN

Cancer cells reroute cellular metabolism toward glycolysis instead of performing oxidative phosphorylation even under aerobic conditions. This is reflected by higher glucose consumption of cancerous tissues referred to as the Warburg effect.^15,16^ To investigate whether SFN impacts cancer cell glucose metabolism, glucose consumption and lactate production were examined in the cell culture medium of SFN-treated PNT2, PC-3, and LNCaP cells. After exposure to increasing SFN concentrations for 7 days, the cell culture medium was collected and analyzed, and the cell viability was investigated in parallel. Our analysis revealed an increase in the consumption of glucose as well as simultaneous lactate production in response to 10 μM SFN (Figure 4A,B). The calculated ratio of produced lactate to consumed glucose confirmed this observation, suggesting activation of glycolysis upon SFN exposure in PNT2, PC-3, and LNCaP cells (Figure 4C).

**Figure 4:**
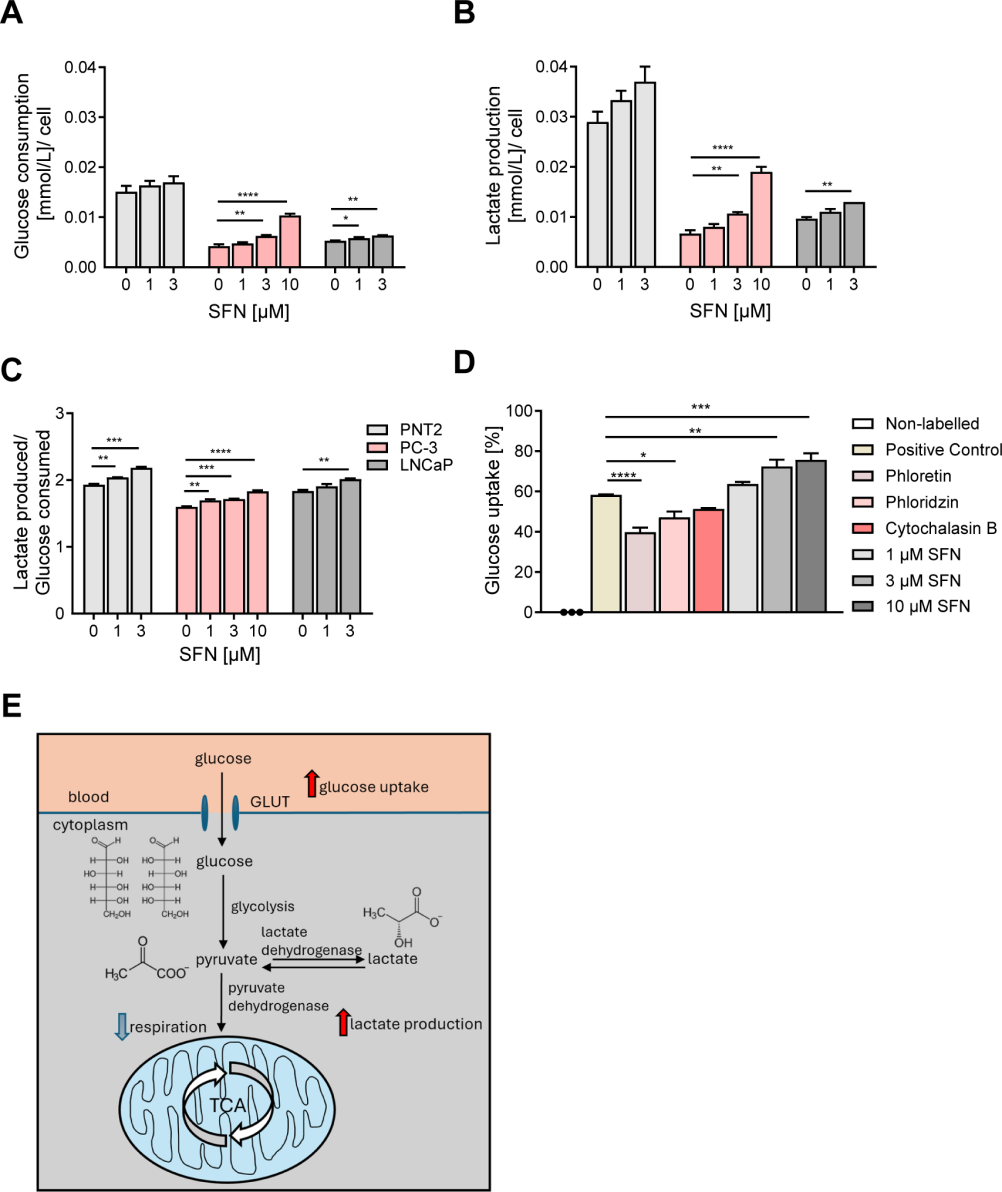
Impact of SFN on prostate epithelial and cancer cell metabolism. (A–C) Medium analysis. PNT2, PC-3, or LNCaP cells was treated for 7 days with SFN (1 μM, 3 μM, 10 μM, and 30 μM) or vehicle (DMSO; 0.1%), and the cell culture medium was collected to be investigated. (A) Glucose consumption; (B) lactate production; and (C) the lactate/glucose ratio. Results were normalized to cell numbers. (D) Glucose uptake assay. PC-3 cells were treated with nonlabeled glucose or 2-(*N*-(7-nitrobenz-2-oxa-1,3-diazol-4-yl)­amino)-2-desoxyglucose (2-NBDG) in combination with vehicle (positive control; DMSO; green), glucose uptake inhibitors phloridzin (100 μM; yellow), phloretin (25 μM; light pink), or cytochalasin B (5 μM; pink) or 1, 3, and 10 μM SFN for 30 min, further stained with PI. Glucose uptake in living (PI-negative) cells was analyzed using flow cytometry. (E) Scheme summarizing SFN impact on glucose metabolism. Data were compared by one-way ANOVA with Dunnett̀s *post hoc* test versus the respective control sample. *n* = 3. *P < 0.05, **P < 0.01, ***P < 0.001, and ****P < 0.001.

This result was corroborated by a glucose-uptake assay using 2-(*N*-(7-nitrobenz-2-oxa-1,3-diazol-4-yl)­amino)-2-desoxyglucose (2-NBDG), a fluorescent glucose analogue (Figure 4D). PC-3 cells were treated either with nonlabeled glucose (white) or with 2-NBDG alone (positive control; green) or 2-NBDG in additional presence of the established glucose uptake inhibitors phloretin (yellow), phloridzin (light pink), or cytochalasin B (pink), or increasing concentrations of SFN (1 μM, 3 μM, and 10 μM; gray bars). The treatment was followed by starvation in a glucose-free medium for 24 h. In contrast to the glucose uptake-inhibitors that revealed significantly reduced uptake of 2-NBDG versus vehicle-treated control cells, SFN-exposure resulted in a significantly increased uptake of 2-NBDG (Figure 4D). The effects of SFN on cancer cell metabolism are summarized in Figure 4E.

Thus, our data suggest that SFN stimulates glycolytic metabolism in both prostate cancer and noncancer cells, potentially as a stress-adaptation and drug-resistance mechanism by stimulating the pentose phosphate pathway and producing more NADPH that may ultimately partially mitigate the anticancer effects of SFN. This metabolic shift could offer additional therapeutic opportunities, such as combining SFN with glycolysis inhibitors or oxidative phosphorylation modulators that may further enhance the cytotoxic activity of SFN in cancer cells. Moreover, coadministration of SFN with glucose-drug conjugates might potentiate the uptake and efficacy of the latter and therefore should be further investigated.

Overall, our results confirm the anticancer effects of SFN. In cancer patients, the plasma levels of SFN—either alone or conjugated to other metabolites—have been demonstrated to reach low micromolar concentrations, ranging from 0.12 to 2.12 μM.^17–19^ This matches the lowest concentration used (3 μM) that we used in this study for long-term exposure experiments, thus representing a clinically relevant scenario within the context of patient treatment. It is also important to note that inside the body, SFN forms protein or glutathione adducts, which make it available to trans-thiolate other proteins and thus prolong its actions and extend its functional effects.^20,21^

### Simultaneous Assessment of SFN Effects on Cancer Cell Apoptosis and hiPSC-CM EHT Contractile Function

Next, we established a human in vitro coculture model to illustrate the anticancer properties of SFN and simultaneously monitor its impact on contractile properties or potential cardiotoxic side effects. HiPSC-CM EHT (*n* = 17) and PC-3 cells were kept in coculture for 7 days, and spontaneous beating frequencies and force development of hiPSC-CM EHT were monitored every other day (Supplementary, Figure 1A). Experiments shown before (Figures 1 and 2) revealed that a long-term applied concentration of 3 μM SFN was successful in achieving prostate cancer cell apoptosis by caspase activation and reduction of colony formation. At day 8 post coculture onset, hiPSC-CM EHT-PC-3 were allocated into 2 force-matched groups receiving vehicle (control; DMSO; black; *n* = 8) or SFN (3 μM; gray; *n* = 9) for 7 days. Spontaneous beating frequency (Supplementary, Figure 1B) and force (Figure 5A) were assessed daily. Cancer cell density was monitored by bright-field microscopy and revealed enhanced cell death of PC-3 cells in response to SFN-exposure compared to vehicle DMSO (Figure 5B). After 7 days exposure to DMSO or SFN, hiPSC-CM EHT was electrically stimulated at increasing frequencies (0.5, 1, 1.5, 2, and 2.5 Hz; Figure 5C), and developed force and average peak force were assessed (Figure 5D,E). There was no difference in developed force between intervention groups (at 2.5 Hz, DMSO: 0.15 ± 0.01 mN vs SFN: 0.15 ± 0.01 mN) detectable. However, hiPSC-CM EHT treated with the vehicle showed impaired ability to follow the electrical pacing signal (Figure 5C) and exhibited an irregular beating pattern. This was evidenced by RR scatter analysis that was performed up to a 4 Hz pacing frequency (Figure 5F, left). In contrast, cocultures of the SFN-treated group revealed time-dependent PC-3 cell death that was paralleled by the ability of hiPSC-CM EHT to follow the electrical beating protocol (Figure 5C,F, right). Examples of irregular beating behavior of vehicle-treated hiPSC-CM EHT (DMSO) in the presence of PC-3 cells are exemplified by the original contraction traces (Supplementary, Figure 1C).

**Figure 5:**
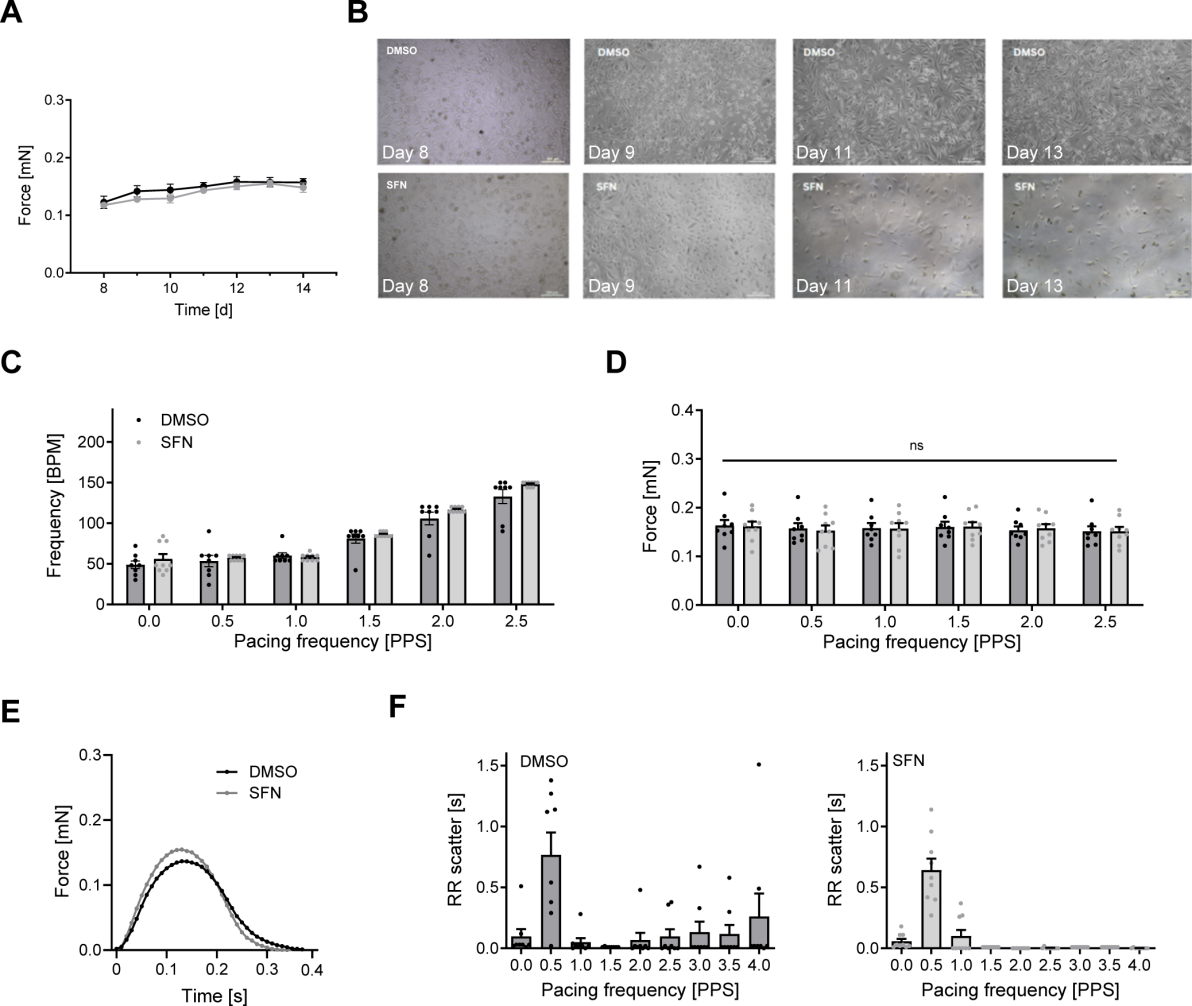
Evaluating the effect of SFN on PC-3 prostate cancer cell survival and simultaneously on hiPSC-CM EHT contractility. (A) HiPSC-CM EHT was cocultured with PC-3 cells. Cocultures were exposed to vehicle (DMSO; 0.1%; black dots; *n* = 8 EHT) or sulforaphane (SFN; 3 μM; gray dots; *n* = 9 EHT) for 7 days, and spontaneous force (mN) development was recorded daily. (B) Brightfield images of cocultured PC-3 cells exposed to vehicle (DMSO) or SFN visualized SFN-mediated cell death versus vehicle-treated cells over time. (C) On day 7, hiPSC-CM EHT (vehicle DMSO group; 0.1%; black dots; SFN group; gray dots) was electrically paced at increasing frequencies (0.5, 1, 1.5, 2, and 2.5 Hz). (D) Force (mN) development was assessed (vehicle DMSO group; black; SFN group; gray). (E) The average force peak for vehicle- (black) and SFN (gray)-treated hiPSC-CM EHT obtained at 2.5 Hz is shown. (F) RR scatter as an indicator of beating regularity at increasing pacing frequencies (0.5, 1, 1.5, 2, 2.5, 3, 3.5, and 4 Hz) for vehicle- (black; left) or SFN (gray; right)-treated hiPSC-CM EHT is shown.

Cardiotoxic side effects of anticancer drugs are a severe and unfortunately common problem in cancer therapy.^22^ We described previously that SFN impacts cardiomyocyte contractility and mitochondrial function in EHT derived from neonatal rat ventricular myocytes and thus might convey cardiotoxic side effects in patients.^8^ To account for direct actions of drugs or cancer cells on cardiomyocytes, we here set out to establish a cost-effective and scalable preclinical human in vitro coculture model combining hiPSC-CM EHT with prostate adenocarcinoma cells in coculture to study SFN effects on cancer cell death and hiPSC-CM EHT contractility simultaneously.

This coculture model is an addition to the plethora of applications of EHT technology that have been proven extremely valuable for applications such as a drug testing platform operating in a convenient 24-well format.^12,23–26^ As such, the EHT culture format has been used previously to explore the mechanisms by which tyrosine kinase inhibitors that are frequently employed in the treatment of cancer patients evoke cardiotoxic side effects.^27^

Taking the 3R principles and the FDA’s modernization act into account, it allows us to study drug effects on a human genetic background and, at the same time, to save animal lives and to expand on the ever-growing spectrum of in vitro models in the cardio-oncology field.^28,29^

The suitability of the herein described in vitro coculture model to simultaneously observe the effects of anticancer drugs on malignant cell proliferation as well as potential adverse side effects on cardiomyocyte contractility was exemplified by testing SFN, a phyto-compound that has been attributed broad anticancer properties.^30^ Indeed, during the 7 day SFN exposure conducted in this study, there was a decline in PC-3 prostate cancer cell number detectable with an unexpected stabilization of the contractile beating pattern of hiPSC-CM EHT compared to the vehicle-treated group. We described previously that short- or long-term SFN exposure impaired mitochondrial and contractile function in ventricular rat EHT.^8^ In the study performed here employing hiPSC-CM EHT in coculture with PC-3 cells, SFN exposure did not further impair contractile function of hiPSC-CM EHT in the presence of cancer cell coculture but induced cancer cell apoptosis. Importantly, although not reaching statistical significance, SFN exposure of hiPSC-CM EHT even slightly improved force development compared to the vehicle-treated coculture visible by the average force peak (Figure 5D,E). The reason for the discrepant observations made in rat versus human EHT could be either species differences or, more likely, the considerable fraction of other cardiac cell types that are present in the heterogeneous neonatal rat EHT such as fibroblasts, endothelial, or inflammatory cells that might also be affected by the broad SFN actions and consequently contribute to the contractile response. The potential contribution of SFN actions on other cardiovascular cells to human EHT functions should be the focus of future studies enrolling multicell-type EHT. Overall, these results support the versatility of this in vitro coculture model in testing the direct effects of anticancer drugs on contractile function. Particularly for SFN, these data might suggest that this compound could be used to combat cancer without incurring further burden on patients’ cardiac function than that induced by cancer itself.

In this study, we demonstrated the applicability of an in vitro coculture model combining hiPSC-CM EHT with cancer cells to investigate cancer drug actions on cardiomyocyte contractility exemplified here for SFN, but applicable for general drug testing. This model can be further expanded on the use of multicell-type EHT thus representing a versatile platform to identify possible side effects of anticancer drugs on cardiomyocyte function.

## Supplementary Material



## Notes

Frequency and force development of hiPSC-CM EHT monitored over time alone or in the presence of cocultured PC-3 cells; coculture with PC-3 cells induced an irregular beating pattern in hiPSC-EHT (PDF)

## #

DiNABIOS Deutschland GmbH, Start-up Laboratories Hamburg, Luruper Hauptstrasse 1, 22547 Hamburg, Germany

## ¶

Institute of Immunology, University Medical Center Schleswig Holstein, Arnold-Heller-Str 3, 24105 Kiel.

## Abbreviations

2-NBDG: 2-desoxy-2-[(7-nitro-2,1,3-benzoxadiazol-4-yl)­amino]-d-glucose
CM: cardiomyocyte
DMSO: dimethyl sulfoxide
EHT: engineered heart tissue
FACS: fluorescence-activated cell sorting
FCS: fetal calf serum
hiPSC: human-induced pluripotent stem cell
ITC: isothiocyanate
KEAP1: kelch-like ECH-associated protein
NFκB: nuclear factor kappa B
Nrf2: nuclear factor erythroid 2
PI: propidium iodide
PPS: pulse per second
SFN: sulforaphane
