# Supplementary material for: Investigating the Anticancer Effects of Sulforaphane in an In Vitro Coculture Model of Prostate Cancer Cells with Engineered Heart Tissue
Source: ACS Pharmacol Transl Sci. 2025 Nov 14;8(12):4399–409. doi: 10.1021/acsptsci.5c00622 (PMC12707262; doi:10.1021/acsptsci.5c00622)
Supplement: Supplementary file 1 [file pt5c00622_si_001.pdf]

Supporting Information

Investigating the anti-cancer effects of sulforaphane in an in vitro co-culture model of prostate cancer cells with engineered heart tissue

Jane In den Birken[1,2], Laura Rathjens[1,2], Hannah Münch[1,2], Tina Rohlfing[3], Konstantina Stathopoulou[1,2], Alexandra Rhoden[1,2,4], Gunhild von Amsberg[3,5], Thomas Eschenhagen[1,2], Sergey Dyshlovoy[3,5,6], Friederike Cuello[1,2,*]

[1]Institute of Experimental Pharmacology and Toxicology, University Medical Center Hamburg-Eppendorf, Martinistrasse 52, 20246 Hamburg, Germany.

[2]DZHK (German Center for Cardiovascular Research), partner site Hamburg/Kiel/Lübeck, University Medical Center Hamburg-Eppendorf, Martinistrasse 52, 20246 Hamburg, Germany.

[3]Department of Oncology, Hematology and Bone Marrow Transplantation with Section Pneumology, Hubertus Wald Tumorzentrum – University Cancer Center Hamburg (UCCH), University Medical Center Hamburg-Eppendorf, 20246 Hamburg, Germany.

[4]Current address: DiNABIOS Deutschland GmbH, Start-up Labs Hamburg, Luruper Hauptstrasse 1, 22547 Hamburg, Germany.

[5]Martini-Klinik, Prostate Cancer Center, University Medical Center Hamburg-Eppendorf, Martinistrasse 52, 20246 Hamburg, Germany.

[6] Laboratory of Biologically Active Compounds, Institute of Science-Intensive Technologies and Advanced Materials, Far Eastern Federal University, 690922 Vladivostok, Russian Federation

Table of contents:

Figure S1.A. Frequency (bpm) and force (nM) of hiPSC-CM EHT PC-3 co-cultures over time

Figure S1.B. Frequency (bpm) of hiPSC-CM-PC-3 co-cultures exposed to DMSO or SFN

Figure S1.C. Examples of contraction traces of hiPSC-CM EHT PC-3 co-cultures exposed to DMSO

***CORRESPONDENCE:**
Friederike Cuello (f.cuello@uke.de)
Institute of Experimental Pharmacology and Toxicology; University Medical Center Hamburg-Eppendorf, Martinistrasse 52; 20246 Hamburg, Germany. Tel.: +49 (0) 40/7410 57204

Supplementary Figure 1

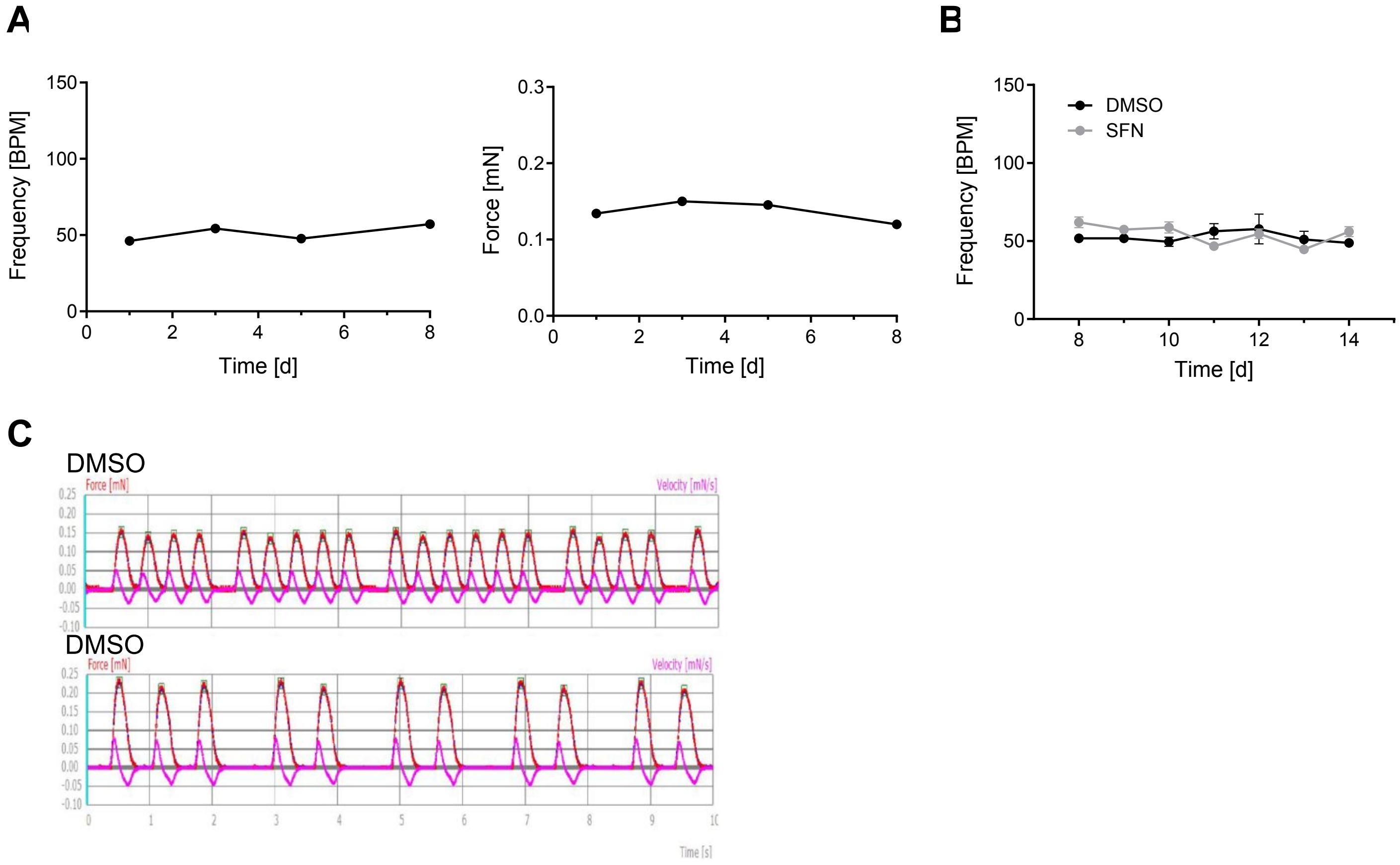

Figure S1. A. HiPSC-CM EHT were co-cultured with PC-3 cells and frequency (BPM; left) and force (mN; right) development assessed every other day. **B.** Co-cultures were exposed to vehicle (DMSO; 0.1%; black dots; n=8 EHT) or sulforaphane (SFN; 3 µM; grey dots; n=9 EHT) for 7 days and spontaneous beating frequency (bpm) was recorded daily. **C.** Examples of irregular vehicle (DMSO)-treated hiPSC-CM EHT contraction traces co-cultured with PC-3 cancer cells are shown.
